# Errors in Abstract

**DOI:** 10.1001/jamanetworkopen.2026.27610

**Published:** 2026-07-15

The Original Investigation titled “Stage IV Breast Cancer Incidence and Survival, 2010-2021,”^1^ published on May 12, 2026, was corrected to fix errors in the Abstract Results. The data reported for increases in percentage of stage IV diagnoses in HR-positive/*ERBB2*-negative, HR-positive/*ERBB2*-positive, HR-negative/*ERBB2*-positive, and triple-negative breast cancer were incorrect. This article was corrected online.^1^
